# The RNA binding protein HuR determines the differential translation of autism-associated FoxP subfamily members in the developing neocortex

**DOI:** 10.1038/srep28998

**Published:** 2016-07-07

**Authors:** T. Popovitchenko, K. Thompson, B. Viljetic, X. Jiao, D. L. Kontonyiannis, M. Kiledjian, R. P. Hart, M. R. Rasin

**Affiliations:** 1 Department of Neuroscience and Cell Biology, Rutgers University, Robert Wood Johnson Medical School, Piscataway 08854, NJ, USA; 2 Department of Cell Biology and Neuroscience, Rutgers University, Piscataway 08854, NJ, USA; 3 Institute of Immunology, Biomedical Sciences Research Center Alexander Fleming, Vari, Greece

## Abstract

Forkhead-box domain (Fox) containing family members are known to play a role in neocorticogenesis and have also been associated with disorders on the autism spectrum. Here we show that a single RNA-binding protein, Hu antigen R (HuR), dictates translation specificity of bound mRNAs and is sufficient to define distinct Foxp-characterized subpopulations of neocortical projection neurons. Furthermore, distinct phosphorylation states of HuR differentially regulate translation of Foxp mRNAs *in vitro*. This demonstrates the importance of RNA binding proteins within the framework of the developing brain and further confirms the role of mRNA translation in autism pathogenesis.

Neurodevelopment relies on specific and timely expression of the genes dictating its course. Transcriptional control is one key mechanism by which appropriate development occurs and manages the expression of fate-determining genes^1^,^2^. Specifically, the transcription factors *Foxp1* and *Foxp2*, members of the Forkhead-box containing (Fox) family, are associated with neocortical development, sensorimotor behavior, and abnormalities such as speech disorders and autism^3^,^4^,^5^,^6^,^7^,^8^.

In addition to transcriptional control, post-transcriptional regulation has emerged as another key mechanism in the spatiotemporal dynamics of brain development. RNA binding proteins (RBPs) control every step of post-transcriptional processing and as such are emerging as fate-determining molecules in neocortical development^9^,^10^. In particular, RBPs such as Fragile X mental retardation protein (FMRP) may regulate a subset of genes associated with the autism spectrum disorders^11^.

Another important family of RBPs is the Hu antigens. Hu antigens (HuA/HuR, HuB, HuC, HuD, and Hel-N1) are named for their role in Hu syndrome, an autoimmune paraneoplastic neurological syndrome, and all have roles in brain development^12^ Mammalian HuR, homologous to ELAV1 (Embryonic Lethal Abnormal Vision) in *Drosophila*, is a globally expressed Hu antigen and has defined roles in brain, breast, lung, and colon^13^,^14^,^15^,^16^. Our group has previously investigated both HuR and HuD RBPs, and revealed an association with neurodevelopmental defects in each case^17^,^18^.

Within the brain, our group previously showed that HuR is an essential regulator of neocortical development and mRNA translation. We found that HuR regulates temporal mRNA association with active translation sites (polysome complexes). This suggests its crucial role in the timing of when a transcript will become a protein in the developing neocortex. However, direct mRNA targets of HuR in neocortical development are unknown and a direct link between HuR and autism has not been observed.

Here, we show that HuR associates with specific and common subsets of mRNAs during early and late phases of prenatal neocortical neurogenesis. The consequence of such differential association is the regulated translational control of *Foxp1* and *Foxp2* at distinct time points in neocortical development. We additionally observed that phosphorylation of distinct HuR sites dictates the dynamic regulation of *Foxp1* and *Foxp2 in vitro*.

## Results

### The RBP HuR binds distinct groups of mRNAs throughout development

**Figure 1:**
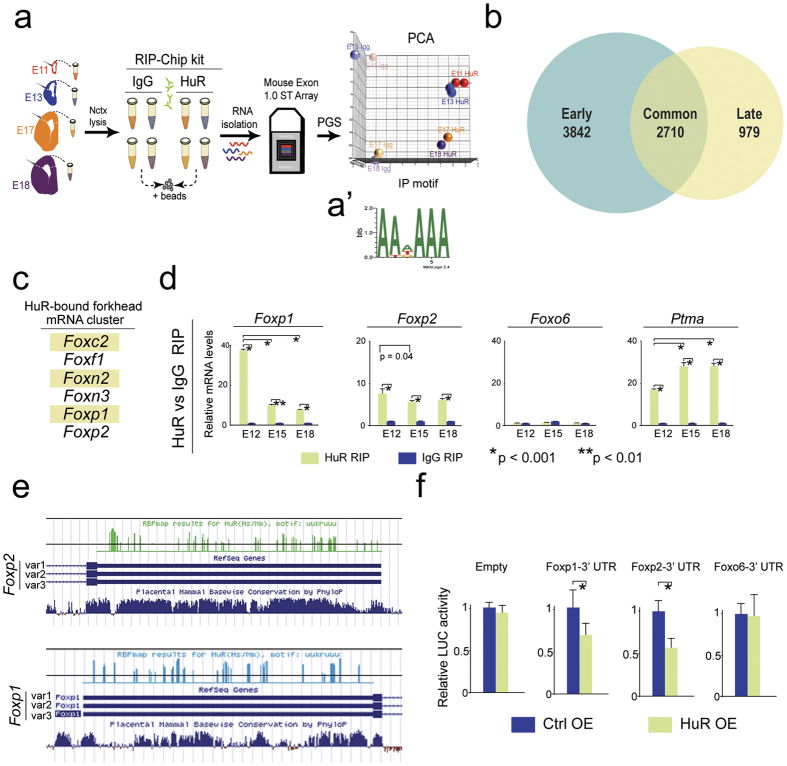
HuR binds *Foxp1* and *Foxp2*, autism-associated Fox mRNAs in developing neocortices. (**a**) Schematic of RIP-ChIP approach from E11, E13, E17 and E18 neocortices to discover HuR-bound mRNAs. RNAs recovered from precipitates were analyzed using Mouse Exon 1.0 ST arrays and PGS or Bioconductor. PCA analysis on the right is shown. (a’) The most prevalent motif of mRNA transcripts in HuR RIP was that of HuR. (**b**) HuR-bound mRNAs were identified during only early (3842 transcripts, blue), or only late (979 transcripts, yellow) neurogenesis, or present in both groups (2710 transcripts, green overlap) during neocorticogenesis. (**c**) HuR binds a group of mRNAs characterized by the Fox domain. (**d**) Additional HuR RIPs from E12, E15, and E18 neocortices followed by qRT-PCR on precipitates validated HuR binding of *Foxp1* and *Foxp2* across neurogenesis. Values were normalized to *Gapdh* and *IgG.* Error bars represent SD, *p < 0.05. (**e**) Predicted HuR binding sites in 3′ UTRs of *Foxp1* and *Foxp2*. (**f**) HuR suppressed luciferase activity when 3′ UTRs of Foxp1 or Foxp2 were present, but not of *Foxo6*.

To elucidate neocortical HuR-bound mRNAs during early neurogenesis (E11 & E13) and late neurogenesis (E17 & E18), we performed RBP-immunoprecipitation coupled to a microarray assay (RIP-Chip) with an HuR RIP-Chip certified antibody and corresponding IgG (Fig. 1a; GEO accession number GSE77712). Principal component analysis (PCA) showed substantial differences in grouping between IgG and HuR pull-downs at distinct developmental stages and clustering among HuR precipitates from neocortices at early (E11 & E13) compared to later (E17 & E18) neurogenesis, substantiating the quality of the RIP experiment (Fig. 1a right).

Using stringent criteria (fold-change > 2 and False Discovery Rate < 5%), we found 6552 transcripts bound in early neocortical neurogenesis, significantly fewer transcripts bound during late neurogenesis (3689), and an overlap of 2710 transcripts bound across these developmental periods (Figs 1b and S1a).

In order to confirm coverage of our screen, we compared our results with a study whose aim it was to determine all binding targets of HuR in HeLa cells. Out of the 1091, 1862, and 564 genes in the early, common, and late lists, respectively, there were 119 (p = 7.92 × 10^−15^), 273 (p = 1.59 × 10^−59^), and 73 (p = 5.29 × 10^−13^) overlaps between our screens. It should be noted that CNS-specific mRNAs which are expressed in murine neocortical tissue may not have been expressed in the HeLa line, accounting for some of the variance between the lists.

We noticed genes associated with autism (members of the FoxP family and DYRK1A) to be HuR-bound and wanted to determine if targets of HuR were enriched for genes related to autism. We did not observe significant overlap between neocortical targets of HuR and genes found in the SFARI autism database, but observed that 12 genes overlap with early, 13 with common, and 2 with late HuR RIP targets; www.sfari.org, Simons Foundation, Table S1). Four genes from this list (NF1, DYRK1A, FOXP1, and GSK3B) were found to be in common with the previous HeLa screen^19^. Additionally, we found a significant overlap of early-bound HuR target genes and those bound by FMRP^9^ (51 genes, Fig. S1b, Table S2). These data suggest that HuR may contribute to regulation of FMRP targets.

### HuR can regulate Foxp1 and Foxp2 via their 3’ UTRs

HuR-bound mRNAs were clustered using functional gene annotation (WebGestalt)^32^, and we found a group of TFs known to be involved in neurogenesis and associated with autism (e.g., Mef2c^33^, Foxp1^4^, Foxp2 4; Fig. 1c, S1b). Subsequent qPCR analyses of additional HuR-RIPs (n = 3 per developmental stage) at E12, E15, and E18 confirmed HuR binding of different members of the *Fox* family (Fig. 1d, S1c). *Foxp1* mRNA was enriched early-on. Meanwhile, *Foxp2* mRNA was bound throughout neocortical neurogenesis, similarly to the known HuR target *Ptma*^20^, and in contrast to the negative control-identified as not bound by HuR in our screen, *Foxo6* (Fig. 1d). HuR did not preferentially bind any splice variant of Foxp1 or Foxp2 (Fig. S1d). Though the expression and roles of Foxp1 and Foxp2 protein in the developing brain are well documented^8^,^23^,^24^,^25^; their upstream regulation has yet to be fully understood.

HuR is known to bind the untranslated regions (UTRs) of mRNAs, which have major roles in post-transcriptional regulation^22^. We found that both *Foxp1* and *Foxp2* had putative HuR binding sites in their 3′ UTRs (Fig. 1e) using RNPmap software (rbpmap.technion.ac.il). We tested for the functional regulation of *FoxP1* or *Foxp2* 3′ UTRs in the presence of HuR using a luciferase assay in HEK293T cells^24^. When normalized over Renilla luciferase, we found that over-expression (OE) of HuR had no appreciable\effect on the expression of empty firefly luciferase vector nor of *Foxo6* 3′ UTR-cloned firefly luciferase (Fig. 1f; n = 3; p > 0.05). However, with either *Foxp1* or *Foxp2* 3′ UTRs cloned downstream of the luciferase open reading frame (ORF), we found a significant reduction in firefly luciferase expression in the presence of the HuR OE, but not the control vector (Fig. 1f; p < 0.05; n = 3). Collectively, these data suggest that HuR may regulate autism-associated *Foxp1* and *Foxp2* expression during neocorticogenesis.

### HuR conditional mutants reveal time-sensitive role of post-transcriptional regulation of Foxp1 and Foxp2

**Figure 2:**
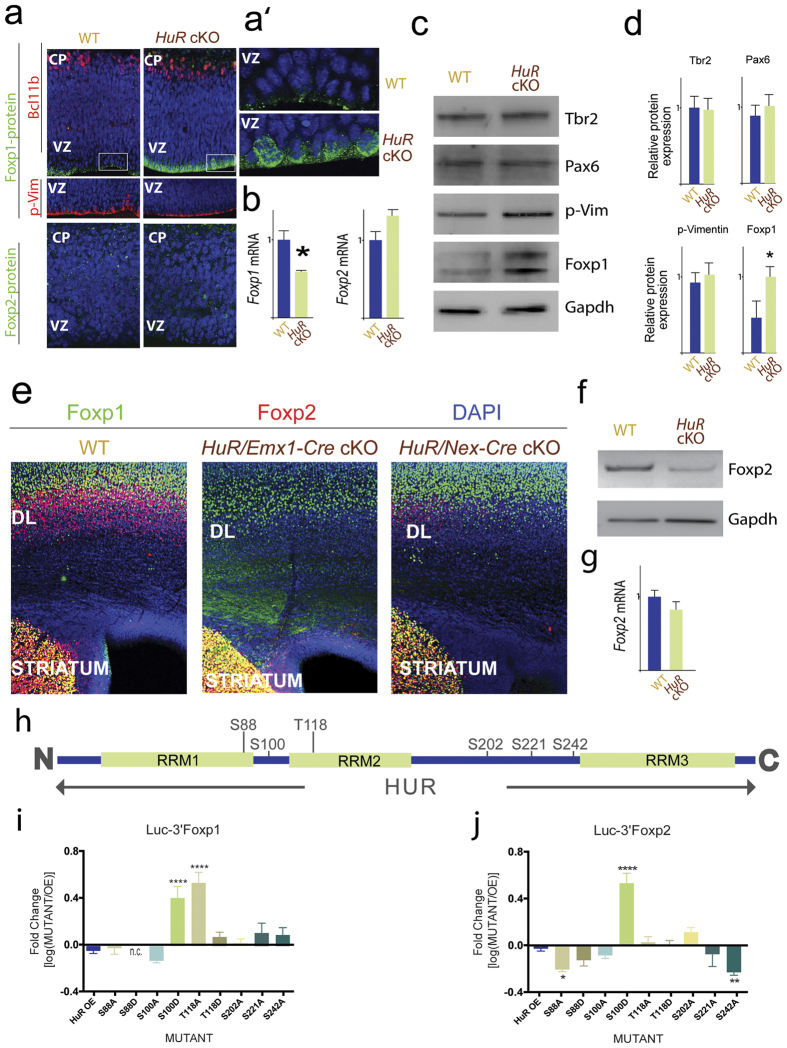
Neocortical HuR-dependent differential regulation of *Foxp1* and *Foxp2* mRNA translation depends on HuR phospho-states. (**a**) Representative confocal images of immunostained E13 neocortex. Foxp1 protein (green, upper panels) was increased in mitotic RG in *HuR* cKOs at E13 and in BCl11b-expressing (red) post-mitotic neurons. Foxp2 protein (green, lower panels) showed no change. Boxed insets of VZ are shown enlarged in (**a’**). (**b**) *Foxp1* mRNA levels were reduced in the E13 *HuR* cKO neocortex, but not *Foxp2*. (**c**) WB analysis confirmed that E13 HuR depletion increased Foxp1 protein, with no detectable changes in Tbr2 or Pax6. Quantification shown in (**d**). Values were normalized to Gapdh. (**e**) Representative confocal images of immunostained P0 neocortex. Foxp2 protein (red) was decreased in neocortices of both neocortical *HuR* cKOs, while being spared in basal ganglia (BG). Foxp1 (green) expression was not lost in *HuR* cKOs. DAPI is in blue. (**f**) WB analysis confirmed decreased levels of protein in HuR cKO, Nex-Cre (**g**) qRT-PCR revealed that levels of *FoxP2* mRNA were unchanged in *HuR* cKO, Emx-Cre (**h**) Schematic of HuR protein with tested Ser/Thr sites. (**i,j**) Translation assay of *Luc-3*′*Foxp1* (**i**) and of *Luc-3*′*Foxp2* (**j**) in the presence of *HuR* OE or a phoshosite-mutated versions. n.c. = not completed Readings represent Luciferase (Luc) protein amount normalized to qRT-PCR measured *Luciferase* (*Luc*) mRNA levels. Graphs are presented as the log_10_ fold change of the *HuR* mutant readings over control OE for Luc/*Luc*. DL = deep layers. Error bars represent SEM, *p < 0.05, **p < 0.01, ****p < 0.0001.

To examine the role of HuR in the post-transcriptional processing of *Foxp1* and *Foxp2* mRNA in the E13 neocortex *in vivo*, we generated an *HuR* conditional knock-out (HuR cKO) line by crossing HuR-floxed mice to Emx1-Cre (*HuR/Emx1-Cre* cKO; HuR depleted in radial glia progenitors and postmitotic neurons.). We performed qRT-PCR on total mRNA isolated from E13 WT and *HuR* cKO littermates and found a significant decrease in *Foxp1* mRNA levels (Fig. 2b; n = 2; p < 0.05). Protein levels in *HuR* cKOs were assessed using immunohistochemistry (IHC) and western blotting (WB) (Fig. 2a,c,d). Surprisingly, despite lower *Foxp1* mRNA levels, we found increased Foxp1-protein expression in RGs at E13, indicating HuR suppression of *Foxp1* translation during early neocortical neurogenesis. When we assessed *Foxp2* mRNA levels, we found them to be unchanged at E13 (Fig. 2b). Furthermore, IHC revealed that Foxp2 protein was not increased in E13 *HuR/Emx1-Cre* cKO neocortices (Fig. 2b), corroborated by WB (data not shown). This suggests distinct temporal regulation of *Foxp1* and *Foxp2* transcripts by HuR.

We next sought to determine if HuR is required for Foxp2 protein expression later in development. IHC of P0 WT, *HuR/Emx1-Cre* cKO, and *HuR/Nex-Cre* cKO (HuR depleted in postmitotic neurons.) neocortices revealed decreased Foxp2 protein expression in both *HuR* cKOs (Fig. 2e, red) and presence of Foxp1 protein at P0 (Fig. 2e, green); WB confirmed decreased Foxp2 protein in *HuR* cKO (Fig. 2f). qRT-PCR confirmed no changes in total *Foxp2* mRNA levels in the HuR cKO (Emx1-Cre, Fig. 2g) nor in subcellular distribution between nucleus and cytoplasm (Emx1-Cre, Fig. S2a). These findings indicate a role for HuR in regulation of Foxp2 mRNA translation. We further determined that HuR was acting in a cell-autonomous fashion (Fig. S2c–h).

### Phosphorylation sites on HuR act in post-transcriptional regulation of Foxp1 and Foxp2

Given HuR’s ability to inhibit translation of *Foxp1* and promote *Foxp2*, we wanted to explore HuR’s capacity to both bind similar targets and temporally regulate them. HuR has multiple residues which are amenable to phosphorylative regulation (Fig. 2h, www.phosphosite.org) and these have been shown to influence HuR’s function as an RBP^28^,^29^. To test this hypothesis, we transfected HuR phosphomutants and phosphomimics along with 3′ UTR *Foxp1-* or *Foxp2*-luciferase constructs in HEK293T cells and performed a translation assay^24^.

For the *Foxp1*-luciferase transfected cells, the phosphomimic S100D and the phosphomutant T118A had significantly higher translation of the luciferase gene than the wild type HuR overexpression (Fig. 2i). In *Foxp2*-luciferase transfected cells, the phosphomimic S100D, as in *Foxp1*, had higher translation than wild type HuR (Fig. 2j). However, in contrast to *Foxp1*, the phosphomutants S88A and S242A had significantly lower levels of *Foxp2* translation than the wild type. This suggests that the S100 site is important to the regulation of translation of both *Foxp1* and *Foxp2* mRNA transcripts, while the S88, T118, and S242 sites contribute to the differential regulation of these two related transcripts.

## Discussion

While there is much known about the downstream targets and effects of Foxp2^28^ few studies have identified the mechanisms behind *Foxp2* or *Foxp1* mRNA regulation. Recently, we identified WNT3 as a positive post-transcriptional regulator of Foxp2, but not Foxp1^24^. Another study examining post-transcriptional regulation of Foxp2 found it to be a target of the miRNAs miR-9 and miR-132^22^. One transcriptional regulator of Foxp2, *lef1,* was previously determined in *Danio rerio* (Zebrafish) to bind an enhancer element of Foxp2 in tectum and hindbrain, but not the telencephalon, a precursory structure to the cerebral cortex^29^. Though these results have yet to be confirmed in higher taxonomic organisms, this is particularly interesting evidence of regulation from an evolutionary perspective. Given this dearth, our study provides needed insight into the regulation of Foxp2.

What is particularly compelling about the regulation of Foxp1 and Foxp2 protein synthesis is the dependence on time. Early neocortical neurogenesis has been known to be susceptible to teratogenic influence that then results in autism-like phenotypes, further underscoring the importance of timing and timed control in brain development^30^,^31^. Our data show the existence of groups of transcripts bound by an RBP, HuR, during early neurogenesis, late neurogenesis, and throughout. Collectively, these data support the idea that some RBP-bound mRNAs form RBP-defined operons^10^,^18^ which are functionally and structurally related mRNAs poised for translation in developing systems, allowing for prompt and rapid response to developmental needs and specification signals. While *Foxp2* mRNA levels did not change in *HuR* cKO, we unexpectedly found decreased *Foxp1* mRNA paralleled by increased Foxp1 protein at E13. This unusual finding may be the consequence of a misbalanced steady state of *Foxp1* mRNA. For example, not enough *Foxp1* mRNA is being transcribed and to compensate for the absence of HuR binding, this mRNA is now being prematurely translated and decayed. As one of many options, this intriguing finding should be further characterized in future studies.

While many studies have shown that HuR can be regulated via its phosphorylation sites, we provide the first evidence that differential HuR phosphorylation states may regulate mRNA translation of the bound autism-associated genes *Foxp1* and *Foxp2*. Sole phospho-sites have been involved in either inhibiting or promoting HuR activity^26^,^34^,^35^,^36^. In addition to the work presented here, one other study showed that tandem phosphorylation of HuR at S221 and S318 was necessary for mRNA binding (COX-2, cyclin-A, and cyclin-D) and nuclear translocation^37^. Our data agree with the hypothesis that the HuR-phosphorylation pattern is responsible for differential translational regulation of bound mRNAs. However, we cannot exclude the possibility that the distinct combinations of phosphorylated sites are further involved in the dynamic temporal regulation of *Foxp1* and *Foxp2* expression levels, with certain sites activated at certain time points in development. While there are some known kinases targeting HuR, including Cdk1(for S202) and PKC α/δ (for S221), no kinase has yet been identified for S242^26^. The precise spatiotemporal mechanism of HuR-phosphosite-mediated regulation as well as which sites are active when *in vivo* are key questions that remain to be answered.

Within the neocortex, Foxp1 and Foxp2 define functionally distinct subpopulations of neocortical glutamatergic projection neurons^23^ and mutations in each are associated with autism^3^,^4^,^5^,^6^,^7^,^8^. This novel mechanism of post-transcriptional regulation demonstrates how intrinsic HuR can differentially regulate translation of bound mRNAs during the intricate steps of neocorticogenesis. In conclusion, this study demonstrates the importance of RBPs within the framework of the developing brain and further confirms that altered mRNA translation contributes to autism pathogenesis.

## Methods

### Animals

All procedures and animal husbandry were approved by and carried out in accordance with the Rutgers University Institutional Animal Care and Use Committee (IACUC) guidelines (protocol number: I12-065). Isolation of wild type (WT) embryonic cortices and *in utero* electroporation experiments were performed in pregnant CD-1 dams (Charles River). Generation of HuR conditional deletion and WT littermate control animals was accomplished using Jackson laboratory Emx1-Cre (Strain Name: B6.129S2-Emx1tm1(cre)Krj/J, Stock Number#: 005628) and *Nex-Cre* (kind gift from M. Schwab). To generate embryonic HuR deletion mice, we produced timed pregnancies by placing a male with females overnight and checking for plugs the next day. The presence of plugs was considered embryonic day 0.5 (E0.5). Neocortical dissections were performed under RNAse free conditions on ice in PBS or Hank’s Balanced Salt Solution (HBSS) supplemented with D-glucose and HEPES^38^. Neocortices were either used immediately or stored at −80 °C until use. At least eight neocortices per the genotype were analyzed in each of the experimental approaches.

### Immunocytochemistry

Brains were dissected in PBS and immediately submerged in 4% paraformaldehyde (PFA) (Sigma-Aldrich #P6148). Three ten-minute washes were carried out at room temperature while agitating and brains were subsequently left agitating in fresh PFA at 4 °C overnight. Fixed brains were washed once with PBS and placed in 30% sucrose (J.T. Baker #4072-05) and stored at 4 °C. Fixed brains were embedded in 3% agarose (Lab Express #2001) and sectioned (Leica Vibratome VT100S) at 70 μm. Sections were stored in 30% sucrose.

For immunostaining, sections were first subjected to three five-minute PBS washes while gently agitating. Next, sections were blocked in PBS with 5% normal donkey serum [Jackson Immuno, 1% bovine serum albumin (BioMatik #A2134), 0.1% glycine (VWR #BDH4156)] containing 0.1% L-lysine (Sigma-Aldrich 100999242) and 0.4% Triton-X (Fisher #BP151) pH 7.5] for 45 minutes at room temperature. After blocking, sections were placed in their respective primary antibodies (Table S3) in blocking solution with 0.4% Triton-X 100 overnight or up to two nights with gentle shaking at 4 °C. Sections were washed in PBS three times and placed in Triton-free blocking solution with secondary antibodies (Table S4) for two hours while gently shaking at room temperature. Sections were then washed three times in PBS for five minutes with gentle shaking and placed in PBS with DAPI (Roche diagnostics #10-236-276-001) for five minutes shaking at room temperature. They were washed again in PBS for five minutes two times. Sections were mounted in VectaShield (Vector Laboratories, Inc. H-1000), cover-slipped, and sealed with nail polish. Imaging was carried out using an Olympus multi-photon/confocal microscope FV1000MPE.

### Western Blot

Protein lysates were prepared using T-PER reagent (Thermo Scientific #78510), Lysates were cleared by centrifugation for 10 minutes at approximately 13,000 × *g*. For loading volume adjustment, lysates were analyzed for total RNA content using a spectrophotometer. We used the Invitrogen SureLock Western blot system with Invitrogen Bis-Tris 4–12% gradient gels and followed the manufacturer’s protocol for running and transfer onto nitrocellulose membranes (BioRad #162-0214).

After transfer, membranes were washed in PBS solution with 0.04% Triton-X 100 (PBS-T), shaking at room temperature for five minutes. Membranes were blocked (blocking solution: 5% milk, 10% FBS, PBS-T) for one hour at room temperature and placed in probing solution (PBS-T and 10% FBS) with primary antibody (see Table S3) overnight or up to two nights, shaking at 4 °C. The blots were washed in PBS solution for five minutes three times, and placed in probing solution with secondary antibody (Table S4) forone1 hour. Blots were washed three times in PBS-T and once in PBS for 5 minutes, and developed (Protein Simple ChemiGlow West #60-12596-00) and imaged with Genesnap software and Syngene G:Box imager. Densitometry quantifications were completed in ImageJ (version:2.0.0-rc-30/1.49u) software.

### Quantitative Real Time PCR (qRT-PCR) and Subcellular Distribution

RNA was isolated using the PARIS kit for cortical and nuclear/cytoplasmic fractionation or Trizol-LS (Life Technologies #10296028), following the manufacturer’s protocol. RNA samples were DNAsed (Ambion #AM1907) to remove contaminating DNA. RT-PCR was performed using Applied Biosystems StepOne Real-Time PCR system with Step-one v2.1 software (#4376373) using RNA-Ct 1-Step Taqman kit (#4392653) and Taqman probes (Table S5) according to the manufacturer’s protocol with reactions adjusted to 10-μl total volume. Each qRT-PCR was performed with more than three technical replicates within more than four biological samples per developmental stage or experimental condition.

### *In utero* Electroporation

E13 neocortices were *in utero* electroporated as previously described^17^,^38^. Briefly, E13 pregnant CD-1 dams (Charles river) were anesthetized, embryos removed, and 1 ug total DNA was injected into the ventricular space using a pulled glass pipette (prepared with Sutter Instrument Co. P-87 pipette puller). Five electric pulses were delivered (BTX Harvard apparatus ECM 830; voltage: 37 V, pulse length: 50 ms, interval between pulses: 950 ms) to embryos by placing the plus electrode above the head to drive DNA into the neocortex. The surgeries lasted approximately twenty-five minutes. Pups were placed back into the mother, muscles and skin were sutured (Ethicon, Silk, 5-0), and gestation was allowed to continue until the appropriate age for subsequent analyses. At least two or three transfected neocortices were analyzed per experimental condition.

### Cell culture conditions and transfections

N2A cells were cultured in Dulbecco’s modified Eagle’s medium (DMEM, GIBCO #31053-028) supplemented with 10% fetal bovine serum (FBS; Sigma-Aldrich #F6178), 20 mM HEPES (GIBCO #15630-080), sodium pyruvate (GIBCO #11360-070), glutamax (GIBCO #35050-061), and penicillin/streptomycin (Cellgro #30-001-C1). HEK293T cells were cultured in DMEM supplemented with 5% fetal bovine serum (FBS; Sigma-Aldrich #F6178), 20 mM HEPES (GIBCO #15630-080), sodium pyruvate (GIBCO #11360-070), glutamax (GIBCO #35050-061), and penicillin/streptomycin (Cellgro #30-001-C1). Both cell lines were fed by aspirating media, washing with PBS 1X and replacing with fresh media. Passage of cells was performed in TrypLE select (GIBCO #12563-029) for five minutes, after which supplemented DMEM media was added at two times the volume of TrypLE and cells were pelleted at 300 rpm for three minutes (Beckman Coulter Allegra 21 centrifuge) and re-suspended in fresh media or lysis buffer for downstream protein/RNA isolation. Transfections were performed using Lipofectamine 2000 (Invitrogen #52758) according to the manufacturer’s protocol.

To prepare for primary neuronal cultures, one day before neocortical dissections, cover slips were treated with 1M HCl and then washed and coated with 15 ug/ml poly-L-ornithine (Sigma-Aldrich #P3655) for forty-five minutes at room temperature. Poly-L-ornithine was aspirated from cover slips that were then treated with 4 μg/ml of mouse laminin (GIBCO #1094706) for 10–15 hours at 37 °C. To isolate primary neurons, neocortical dissections were performed in HBSS supplemented with 0.5% D-Glucose (American Analytical #AB00715-00500) and 25 mM HEPES (GIBCO #15630-080). Dissected neocortices were minced and dissociated in HBSS supplemented with 0.5% D-Glucose, 25 mM HEPES, 0.5 mg/ml DNase I (Sigma-Aldrich #D5025), 20 U/ml Papain (Calbiochem #5125), and 5.0 mg/ml Dispase II (Sigma-Aldrich #D4693-1G). Cells were incubated for five minutes, pelleted, and re-suspended in supplemented HBSS three times. Primary dissociated cells were re-suspended in primary cell culture media including Neurobasal medium (GIBCO #1097077), Sodium Pyruvate (GIBCO #11360-070), 2 mM glutamax (GIBCO #35050-061), penicillin/streptomycin (Cellgro #30-001-C1), and B-27 supplement (GIBCO #17504). All cells were cultured at 37 °C with 5% CO2. For immunostaining, cells were fixed with 4% PFA for twenty minutes.

### HuR RIP

HuR-associated RNAs from neocortices at distinct ages (E11, E12, E13, E15, E17, E18, P0) were isolated using the RIP kit (MBL international #RN1001) and rabbit anti-HuR (MBL international #RN004P) RIP certified antibody according to manufacturer’s instructions. HuR function was shown to react to UV^39^,^40^ used in HITS CLIP, which is avoided by the RIP-ChIP kit.

Previously, targets of HuR were identified in a human HeLa cell line using the PAR-CLIP method. To compare our findings, we first converted their list of target human gene symbols (Supplementary Table 2) to mouse gene symbols. Starting with 1216 human genes, we used the biomaRt tool within R to query the Ensembl database and found the mouse homolog associated gene name for each. With some imprecision in matching for species, this produced 1214 results, but only 1192 unique mouse gene symbols. From these, we then used the GeneOverlap package in R to apply Fisher’s exact test for significance of the overlap between lists of gene symbols from our RIP-ChIP screen and the PAR-CLIP one.

### Microarray analysis

Total RNA from developing neocortices and RIPs were sent for exon microarray hybridization at the UMDNJ-RWJMS facility using the GeneChip Mouse Exon 1.0 ST Array (Affymetrix; n = 2/developmental stage or experimental condition). The Array image (CEL) files were read into either Partek’s Genome Suite (Affymetrix) or R/BioConductor using the oligo package^41^ RMA normalized, and assembled into core transcript models using NetAffx-supplied annotations. A linear model was fit with the limma package^42^ and enrichment contrasts selected based on a log_2_ fold-change of +1 or greater and a Benjamini-Hochberg-adjusted p value of 0.05 or less. Early (E11 and E13) and late (E17 and E18) samples were grouped separately. Of the 4,092 and 2,604 transcripts found to be significantly enriched by HuR RIP at early and late times, respectively, there were 2,244 and 1,803 unique, assigned Entrez Gene IDs and so these were selected for functional analysis using WebGestalt^32^. These transcripts were consolidated into gene symbols for testing overlaps with FMRP-binding mRNAs or autism-associated mRNAs (Fig. S1) using the GeneOverlap package in Bioconductor. Probeset and exon plots were produced using the GenomeGraphs package in Bioconductor.

### Cloning and Constructs

The full-length of Foxp2 3′ UTR was amplified by PCR using the forward primer 5′-TCGCGACTAGTGAACGAACTTGTGACACCTCAGTG-3′ and reverse primer 5′-CATATGCGGCCGTGTACTTCAGAAATGTAACCAACTG-3′. The Foxp1 3′ UTR was amplified with the forward primer 5′-TCGCGACTAGTACATGGAGTGAACCTCTGGGC-3′ and reverse primer 5′-CATATGCGGCCGCATTTAAGAATGCGCTCATGTCAG-3′. Restriction enzyme sites Spe I and Eag I were added to each of the forward primers and reverse primers, respectively, and used for cloning the Foxp2 and Foxp1 3′ UTRs downstream of a firefly Luciferase cassette in the pcDNA3.1-Luciferase plasmid which digested with Xba I and Eag I^24^.

HuR wild type, phosphomimics, and phosphomutants were cloned with TAP tags (Generously shared by Myriam Gorospe tested mutants are indicated in Fig. 2G 34,43). Mutants (S88, S100, T118, S200, S202, and S242) were generated by site-directed mutagenesis of serine/threonine residues to alanine (S/T → A); mimics (S88, S100, and T118) were generated with their serine/threonine residues mutated to aspartic acid (S/T → D).

### Translational Luciferase Assay

To test the effects of these phosphorylation sites on Foxp1 and Foxp2 translation, we performed double transfections with the phosphomutants, phosphomimics, and Foxp1 or Foxp2 with firefly luciferase proteins in their 3′ UTR regions IN HEK293T cells^24^. At least six biological replicates and two technical replicates were performed for each sample; HuR-TAP was biologically replicated at least twenty-four times. Luciferase readings were taken to determine protein expression of the Foxp1/2 constructs (Luc) on a TD-20/20 Luminometer (Turner Designs) and using the Dual-Luciferase Reporter Assay (Promega E1910). Renilla readings were taken as a control to ensure comparable cell counts. Luciferase reagents were made fresh and came from the same kit^44^.

Additionally, RNA was isolated from these samples and quantified with qRT using a luciferase probe (*luc*) (Table S5). Using these two readings (Luc/*luc*), protein and mRNA levels in each sample, we were able to generate a proxy for translation by normalizing protein levels to levels of mRNA^24^. A metric for measuring translation is useful, as levels of mRNA and protein are both informative points of information to the actual state of the cell. For example, in relativity to each other, high levels of mRNA and low protein levels indicate low translation whereas low levels of mRNA and high levels of protein indicate high translation. Data is presented in Fig. 2h,i as their log_10_ values, as we are generating a ratio of mutant translation levels over wild-type translation levels. Outlier analysis was determined by the ROUT test with Q = 1% and the data was subsequently analyzed without the outlier. 6 outliers were removed from the Foxp1 assay (Fig. 2h) out of 160 data points (3.8%) and 10 outliers were removed from the Foxp2 assay (Fig. 2i) out of 172 data points (5.8%).

### Statistical analysis

One-way ANOVA and SPSS software were used for statistical analysis when multiple conditions were compared, while t-test was used when only two experimental conditions within a developmental stage were compared, unless otherwise indicated. N of each experiment and the respective p-values are indicated in either figure legend, results, methods, or in the main text. A Kruskal-Wallis Multiple Comparisons test was performed on data represented in Fig. 2h,i and was calculated in GraphPad’s Prism 6.0 software. A two-way ANOVA was used to compare cultured neurons represented in Fig. S2f (n = 975 neurons, 4 slides, 8 coverslips) and S2g (n = 1082 neurons, 4 slides, 8 coverslips). Scoring and statistical analysis was performed by a blinded experimenter.

## Additional Information

**How to cite this article**: Popovitchenko, T. *et al*. The RNA binding protein HuR determines the differential translation of autism-associated FoxP subfamily members in the developing neocortex. *Sci. Rep.* **6**, 28998; doi: 10.1038/srep28998 (2016).

## Supplementary Material

Supplementary Information
