# Supplementary material for: The RNA binding protein HuR determines the differential translation of autism-associated FoxP subfamily members in the developing neocortex
Source: Sci Rep. 2016 Jul 7;6:28998. doi: 10.1038/srep28998 (PMC4935837; doi:10.1038/srep28998)
Supplement: Supplementary Information [file srep28998-s1.pdf]

The RNA binding protein HuR determines the differential translation of autism-associated FoxP subfamily members in the developing neocortex

Popovitchenko T[1], Thompson K[1], Viljetic B[1], Jiao X[2], Kontonyiannis DL[3], Kiledjian M[2], Hart RP[2], Rasin MR[1,+]

[1]Department of Neuroscience and Cell Biology, Rutgers University, Robert Wood Johnson Medical School, Piscataway, 08854, NJ, USA

[2]Department of Cell Biology and Neuroscience, Rutgers University, Piscataway, 08854, NJ, USA

[3]Institute of Immunology, Biomedical Sciences Research Center Alexander Fleming, Vari, Greece

[+] corresponding co-author:

E-mail: roko.rasin@rutgers.edu

Phone: 1-732-235-4553

Supplementary Figure Legends

Figure S1. HuR binds a subset of Fox mRNAs in developing neocortices. (a) Volcano analysis of HuR RIP-ChIPs identified transcripts selected for a 5% FDR and at least 2-fold enrichment over IgG control. **(b)** Odds ratio for overlapping gene symbols between early and late HuR RIP-ChIP identified transcripts and those reported to bind FMRP[4] or those associated with Autism (http://gene.sfari.org). **(c)** Additional RIPs followed by quantitative RT-PCR analysis of HuR and IgG precipitates confirmed binding of the remaining forkhead mRNAs across neocorticogenesis. Error bars represent SD. *p<0.05. **(d)** HuR did not preferentially precipitate any splice variant of *Foxp1* or *Foxp2*. Array data were plotted to the indicated genome regions using the GenomeGraphs package in R/Bioconductor.

Figure S2. Figure S6. HuR cell-autonomously regulates neocortical expression of FOXP2 protein. (a) *HuR* deletion did not disrupt nuclear-cytpolasmic distribution within P0 neocortices in Emx-Cre animals. **(b)** Successful knockdown of HuR protein using HuR shRNAs. N2a cells were transfected for 72 hours with either Ctrl or HuR shRNAs. Subsequent immunoblotting analysis revealed significant reduction in HuR protein expression with HuR shRNA but not with Ctrl. GAPDH was used as a loading control. **(c)** *In utero* electroporation (IUE) of vectors expressing *Green fluorescent protein* (*Gfp*) and either control (Ctrl) shRNA or *HuR* shRNA was performed at E13 and examined at E18. GFP-positive neurons (green) expressing Ctrl shRNA showed significant HuR expression (red; overlap seen in yellow; left panel) whereas little to no HuR signal was seen in GFP-positive neurons expressing HuR shRNA (middle panel). HuR protein expression was rescued when HuR shRNA was co-electroporated with human HUR cDNA (right panel), indicating specificity of HuR shRNA. **(d)** Silencing of HuR resulted in significant decrease of FOXP2 protein expression (p<0.01). When HuR silencing was rescued using human HuR cDNA, the expression of FOXP2 protein was also rescued. **(e)** Schematic of experimental approach in **(f,g)** co-IUE of E13 neocortices with Ctrl shRNA/Gfp or *HuR* shRNA/Rfp constructs

in same litter. Primary neuronal cultures prepared from pooled electroporated neocortices were analyzed after 5 DIV by immunohistochemistry for Foxp2 in transfected cells for primary neuron experiment shown in **(f,g)**. Representative images show Foxp2 staining (blue) in neurons co-electroporated with HuR shRNA (green) and control shRNA (red). **(h)** Quantifications of confocal images revealed significant decrease of Foxp2 positive neurons in *HuR* shRNA/Gfp neurons when compared to Ctrl shRNA/Rfp neurons. Error bars represent SEM, ****=p<0.0001.

Supplementary Table Legends

Table S1. Overlapping gene targets of HuR and SFARI database: Shown in three columns, left to right, early, late, and common.

Table S2. Overlapping gene targets between RBPs FMRP and HuR at early stages (E11 & E13)

Table S3. Primary antibodies used

Table S4. Secondary antibodies used

Table S5. qRT-PCR probes used

Figure S1

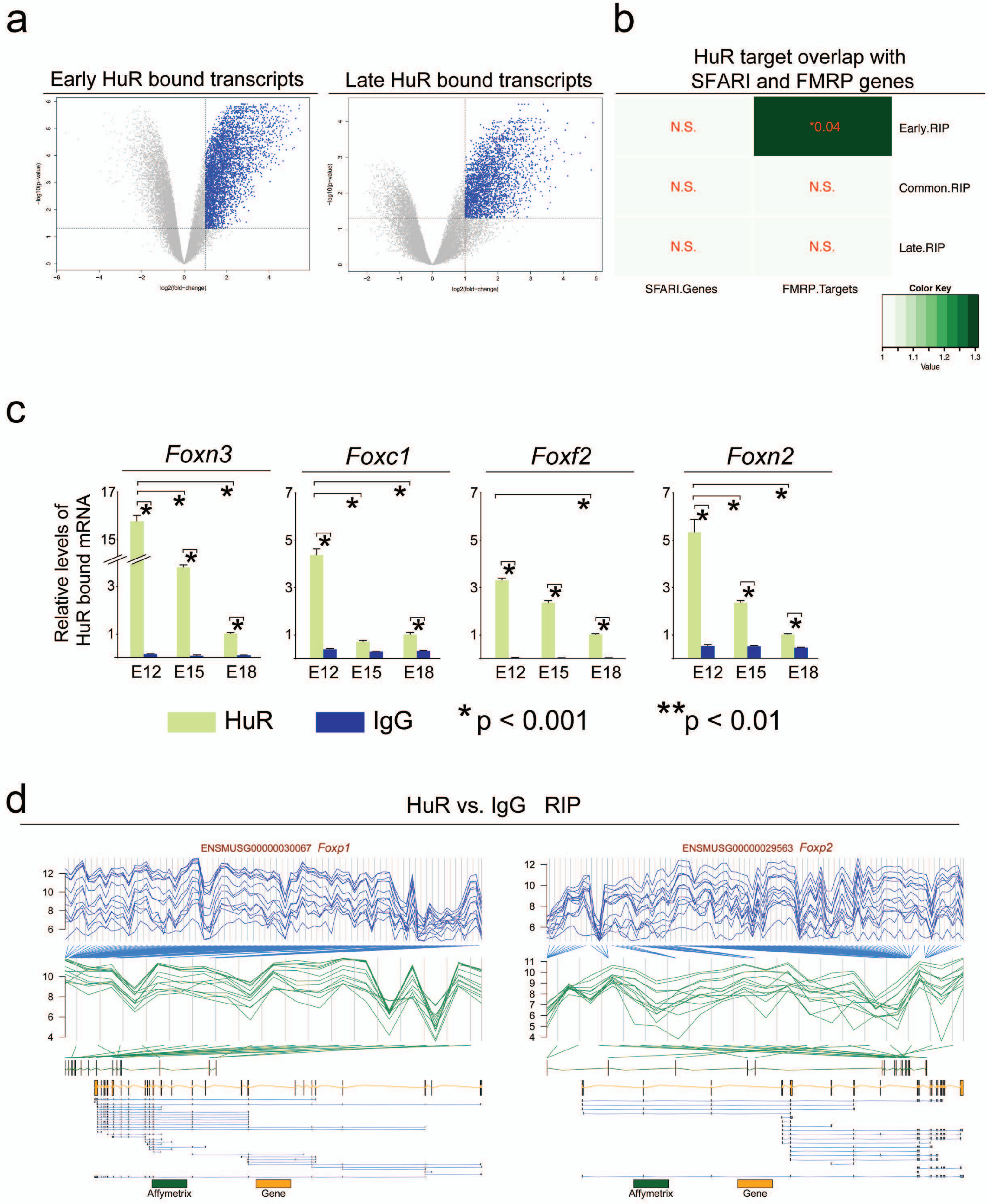

a
Early HuR bound transcripts
Late HuR bound transcripts
-log10(p-value)
log2(fold-change)
b
HuR target overlap with SFARI and FMRP genes
N.S.
*0.04
Early.RIP
Common.RIP
Late.RIP
SFARI.Genes
FMRP.Targets
Color Key
Value
c
Foxn3
Foxc1
Foxf2
Foxn2
Relative levels of HuR bound mRNA
E12
E15
E18
HuR
IgG
* p < 0.001
** p < 0.01
d
HuR vs. IgG RIP
ENSMUSG00000030067 Foxp1
ENSMUSG00000029563 Foxp2
Affymetrix
Gene

Figure S2

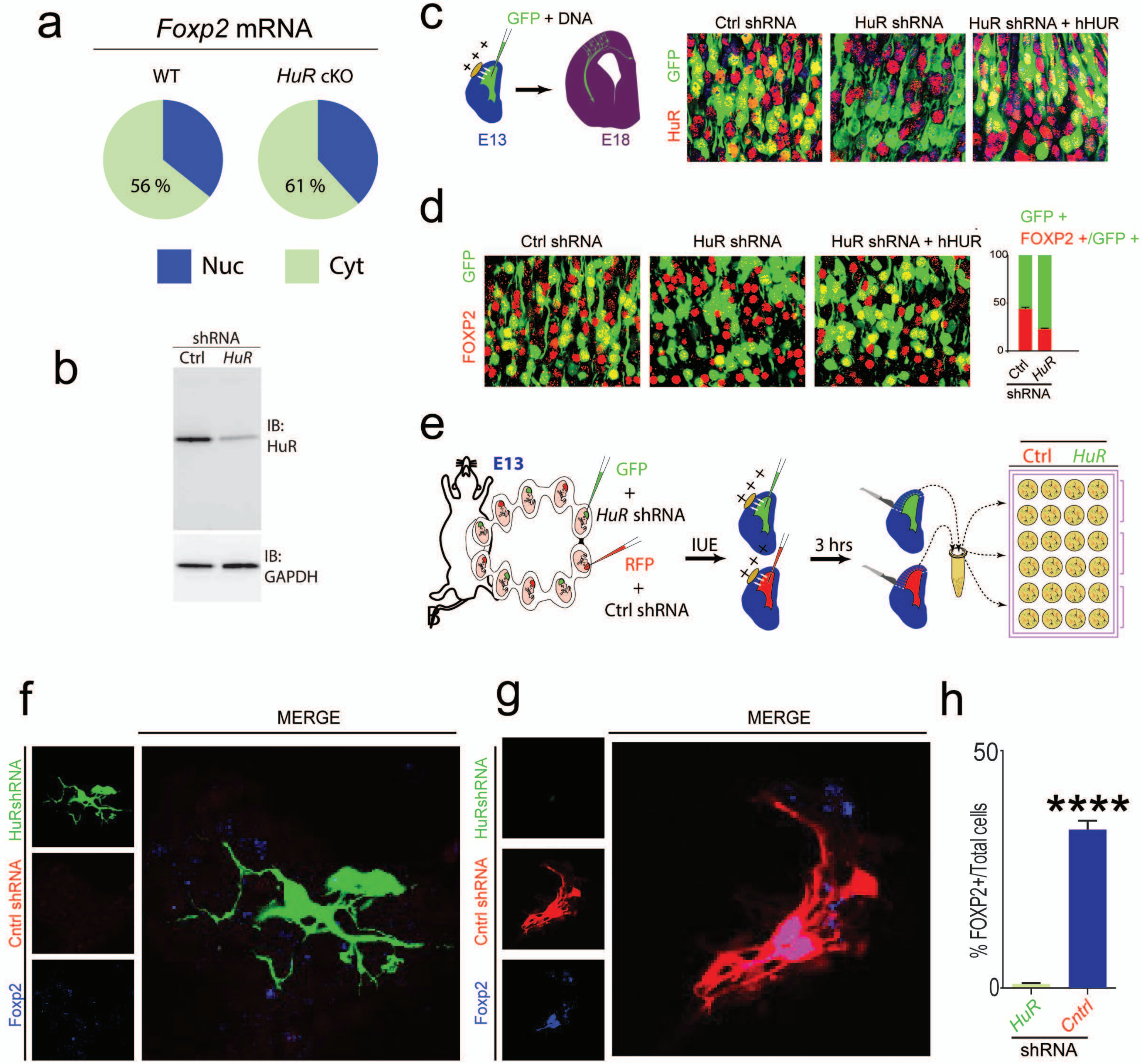

a
Foxp2 mRNA
WT
HuR cKO
56 %
61 %
Nuc
Cyt
b
shRNA
Ctrl
HuR
IB: HuR
IB: GAPDH
c
GFP + DNA
E13
E18
Ctrl shRNA
HuR shRNA
HuR shRNA + hHUR
GFP
HuR
d
Ctrl shRNA
HuR shRNA
HuR shRNA + hHUR
GFP
FOXP2
GFP +
FOXP2 +/GFP +
100
50
0
Ctrl
HuR
shRNA
e
E13
GFP
+
HuR shRNA
RFP
+
Ctrl shRNA
IUE
3 hrs
Ctrl
HuR
f
MERGE
HuRshRNA
Cntrl shRNA
Foxp2
g
MERGE
HuRshRNA
Cntrl shRNA
Foxp2
h
50
0
% FOXP2+/Total cells
****
HuR
Cntrl
shRNA

Table S1: Overlapping gene targets of HuR and SFARI database

Early	Common	Late
ALDH5A1	CADM1	APC
ANK2	CD38	SLITRK5
CADPS2	CYFIP1	
DAB1	DHCR7	
FOXP2	DLG4	
MEF2C	DRYK1A	
NF1	FMR1	
NRG1	FOXP1	
PAFAH1B1	GSK3B	
PCDH10	HOMER1	
	NLGN3	
	PTEN	
	UBE3A	

Table S2: Overlapping gene targets of HuR and FMRP

1110012J17RIK	DPP8	NCAM1	TNKS
1110018G07RIK	DPYSL2	NF1	TNRC6B
1200009O22RIK	EHMT1	NUP98	TRIO
4921513D23RIK	HERC1	PCDH10	TRRAP
ACTB	HIPK1	PDE4DIP	TULP4
ANK2	HIPK3	PPARGC1A	USP9X
ARHGEF11	INPP4A	PTPRD	ZFP462
BMPR2	LPHN3	SAPS2	ZFP521
C230096C10RIK	LRRC41	SASH1	ZFP536
CALM1	MED13L	SCD2	
CALM3	MIB1	SLC12A6	
DIP2B	MKL2	SLC8A1	
DLG5	MTMR4	STXBP1	
DOPEY1	MYO10	TCF4	

Table S3: Primary Antibodies

Name	Species	Cat. #	Supplier	[Conc.]	Dilution	Use
Anti-ELAV1/HUR	Rabbit	RN004P	MBL	1 mg/mL	1:500	WB
Bcl11b (25B6)	Rat	SC-56014	Santa Cruz	100 µg/mL	1:100	IHC
Foxp1	Rabbit	AB16645	Abcam	1 mg/mL	1:1000	IHC
Foxp1	Mouse		Tucker Lab		1:1000	WB
Foxp2 (N-16)	Goat	SC-21069	Santa Cruz	100 µg/mL	1:250<br>1:500	IHC, ICC<br>WB
Gapdh	Mouse	MAB374	Millipore	1 mg/mL	1:5000	WB
HuR	Mouse	39-0600	Invitrogen		1:250<br>1:500	IHC<br>WB
HuR (19F12)	Mouse	SC-56709	Santa Cruz	100 µg/mL	1:1000<br>1:500	IHC<br>WB
HuR (N-16)	Goat	SC-5483	Santa Cruz	200 µg/mL	1:1000	
P-Vimentin (S56)	Rabbit	3877S	Cell Signaling	1 mg/mL	1:100	IHC
P-Vimentin	Mouse	D076-3	MBL	1 mg/mL	1:250	WB
Pax6	Rabbit	PRB-278P	Covance	2 mg/mL	1:500	WB

Table S4: Secondary Antibodies

Name	Fluorophore/ Conjugate	Cat. #	Supplier	Dilution
Donkey anti-Goat	488	705-485-147	Jackson Immuno Research	1:250
Donkey anti-Goat	Cy3	705-165-147	Jackson Immuno Research	1:250
Donkey anti-Goat	HRP	705-035-147	Jackson Immuno Research	1:5000
Donkey anti-Goat	HRP	705-035-003	Jackson Immuno Research	1:5000
Donkey anti-Mouse	Cy3	715-165-151	Jackson Immuno Research	1:250
Donkey anti-Mouse	Cy5	715-605-151	Jackson Immuno Research	1:250
Donkey anti-Mouse	HRP	715-035-150	Jackson Immuno Research	1:5000
Donkey anti-Rabbit	488	715-545-152	Jackson Immuno Research	1:250
Donkey anti-Rabbit	HRP	711-035-152	Jackson Immuno Research	1:2500
Donkey anti-Rat	649	712-495-153	Jackson Immuno Research	1:250

Table S5: qRT-PCR Probes

Name	Mm. #	Lot	Company
FoxC1	Mm01962704_s1	761452	Applied Biosystems
FoxF2	Mm00515793_m1	487722	Applied Biosystems
FoxN2	Mm00839106_g1	454413	Applied Biosystems
FoxN3	Mm02343199_g1	574053	Applied Biosystems
FoxO6	Mm00809934_s1	499576	Applied Biosystems
FoxP1	Mm00474845_m1	722413	Applied Biosystems
FoxP2 FAM long	Mm01161745_m1	P120613-001 H12	Applied Biosystems
Foxp2 FAM pan	Mm00475031_m1	P120613-001 H11	Applied Biosystems
GAPDH	Mm03302249_g1	1067721	Applied Biosystems
PTMA	Mm02342431_g1	716417	Applied Biosystems
